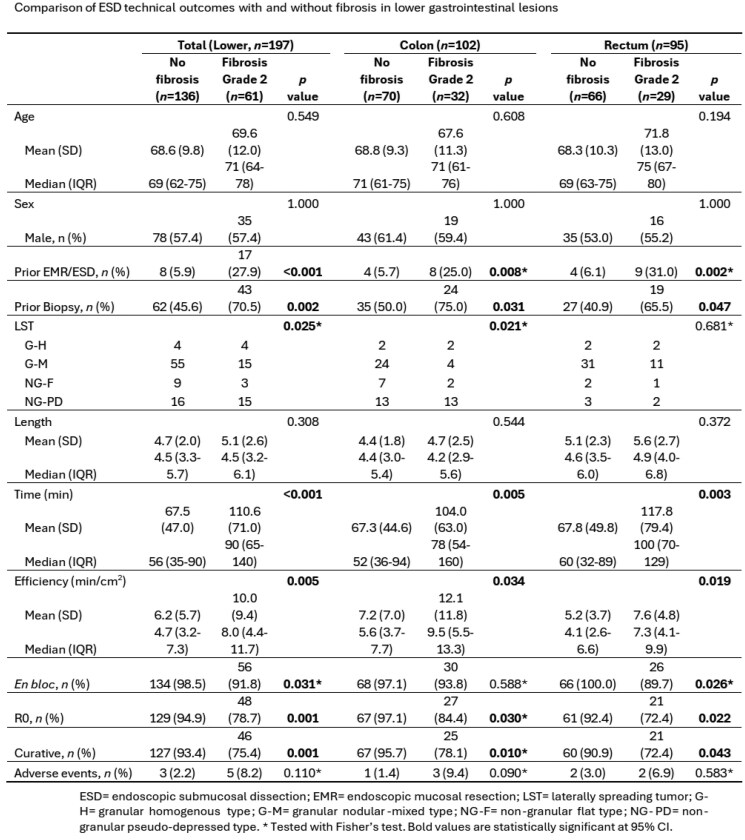# Poster Session I - A160 ROLE OF PREPROCEDURAL BIOPSIES IN LOWER GASTROINTESTINAL TUMORS UNDERGOING ENDOSCOPIC SUBMUCOSAL DISSECTION (ESD) AND IMPACT ON FIBROSIS AND RESECTION RATES

**DOI:** 10.1093/jcag/gwaf042.160

**Published:** 2026-02-13

**Authors:** M Youssef, C Ching Hui Yee, S Ghobrial, K Khalaf, R Bechara

**Affiliations:** Gastroenterology & Hepatology, University of Toronto, Toronto, ON, Canada; McGill University, Montreal, QC, Canada; University of Manitoba, Winnipeg, MB, Canada; Division of Gastroenterology, St. Michael’s Hospital, University of Toronto, Toronto, ON, Canada; Kingston Health Sciences Centre, Kingston, ON, Canada

## Abstract

**Background:**

Endoscopic submucosal dissection (ESD) allows for curative resections of complex colorectal lesions which have high risk of malignancy. Although preprocedural biopsies are routinely done, their role is limited due to sampling error and the associated risk of fibrosis which may hinder dissection and compromise technical outcomes.

**Aims:**

This study aims to evaluate the association between preprocedural biopsies of colorectal lesions and the associated risk of fibrosis and its impact on ESD technical outcomes.

**Methods:**

This is a retrospective cohort study of all patients who underwent ESD for colorectal lesions at Kingston Health Sciences Center (KHSC) between October 2016-September 2025. We analyzed clinical, endoscopic, and histopathologic characteristics of lesions as well as patient outcomes. Histological diagnoses were categorized using the WHO Classification criteria for GI lesions, 5th edition. Rates of concordance and upstaging were calculated comparing the initial and final resected pathology. Univariate analysis between the presence of fibrosis and technical outcomes such as resection time, technical efficiency, en-bloc, R0, curative resections, and adverse events were used to calculate odds ratios with 95% confidence intervals. P-value of < 0.05 was considered statistically significant.

**Results:**

This cohort study included 197 patients with lower GI lesions (colon=102, rectal= 95) that underwent ESD at KHSC. 105/197 (53.2%) of patients had preprocedural biopsies prior to their ESD at KHSC. The overall histologic concordance rate between tissue biopsy and ESD specimens was 34.0% (30.9% for colon and 37.8% for rectal). 64.0% (n = 64) of lesions were upstaged from initial biopsy with 45.3% (n = 29) of those final resections being neoplastic lesions. Lesions with initial biopsies had significantly greater rates of fibrosis (41.0% vs 19.6%, p = 0.001). Patients with prior EMR/ESD at a different center also had higher rates of fibrosis (70.5% vs 45.6%, p < 0.001). Lesions with fibrosis were more likely to have less efficient procedures (mean 10.0 min/cm^2^ vs 6.2 min/cm^2^, p = 0.001). The presence of fibrosis was significantly associated with lower rates of en-bloc resections (91.8% vs. 98.5%, OR 0.18, p = 0.03), R0 resections (78.7% vs. 94.9%, OR 0.20, p = 0.001), and curative resections (75.4% vs. 93.4%, OR 0.22, p = 0.001) but no increased risk of adverse events (8.2% vs 2.2% p = 0.11). Subgroup analyses revealed similar trends when analyzing colon and rectal lesions separately.

**Conclusions:**

Preprocedural biopsies have poor concordance rate with post-ESD tissue histology. Biopsies may increase the risk of fibrosis which may decrease the rates of en-bloc and curative resections. Larger studies should evaluate the incremental benefit of biopsies and their potential associated risks.

**Funding Agencies:**

NoneUniversity of Toronto - Gastroenterology & Hepatology Department